# Clinical Characteristics, Management, and In-Hospital Mortality in Patients with Heart Failure with Reduced Ejection Fraction According to Sex and the Presence of Type 2 Diabetes Mellitus

**DOI:** 10.3390/jcm11041030

**Published:** 2022-02-16

**Authors:** Manuel Méndez-Bailón, Noel Lorenzo-Villalba, Rodrigo Jiménez-García, Valentin Hernández-Barrera, Jose María de Miguel-Yanes, Javier de Miguel-Diez, Nuria Muñoz-Rivas, Emmanuel Andrès, Ana Lopez-de-Andrés

**Affiliations:** 1 Internal Medicine Department, Hospital Clínico San Carlos, Universidad Complutense de Madrid, Instituto de Investigación Sanitaria del Hospital Clínico San Carlos (IdISSC), 28040 Madrid, Spain; manuelmenba@hotmail.com; 2 Service de Médecine Interne, Diabète et Maladies Métaboliques, Hôpitaux Universitaires de Strasbourg, 67000 Strasbourg, France; emmanuel.andres@chru-strasbourg.fr; 3 Department of Public Health & Maternal and Child Health, Faculty of Medicine, Universidad Complutense de Madrid, 28040 Madrid, Spain; rodrijim@ucm.es (R.J.-G.); anailo04@ucm.es (A.L.-d.-A.); 4 Department of Preventive Medicine and Public Health, Faculty of Health Sciences, Universidad Rey Juan Carlos, Alcorcon Madrid, 28922 Madrid, Spain; valentin.hernandez@urjc.es; 5 Internal Medicine Internal Medicine Department, Hospital General Gregorio Marañon, 28007 Madrid, Spain; josemaria.demiguel@salud.madrid.org; 6 Respiratory Department, Hospital General Universitario Gregorio Marañón, 28007 Madrid, Spain; javier.miguel@salud.madrid.org; 7 Internal Medicine Department, Hospital Universitario Infanta Leonor, 28031 Madrid, Spain; nmrivas@hotmail.com

**Keywords:** sex, diabetes mellitus, heart failure with reduced ejection fraction, mortality

## Abstract

Background: Type 2 diabetes mellitus (T2DM) is a risk factor for the development of heart failure with reduced ejection fraction (HFrEF). Aims: (1) To describe and compare the clinical characteristics and the use of diagnostic and therapeutic procedures among subjects hospitalized with HFrEF according to the presence of type 2 diabetes mellitus (T2DM) and sex; (2) to assess the effect of T2DM and sex on hospital outcomes among the patients hospitalized with HFrEF using propensity score matching (PSM); and (3) to identify which clinical variables were associated to in-hospital mortality (IHM) among the patients hospitalized with HFrEF and T2DM according to their sex. Methods: A retrospective cohort study from 2016 to 2019 using the Spanish National Hospital Discharge Database was conducted. The diagnosis and procedures were codified with the International Classification of Disease 10th version (ICD10). Subjects aged ≥ 40 with a primary diagnosis of HFrEF were included. We included those patients with a diagnosis of T2DM in any diagnosis position. The descriptive statistics used were total and relative frequencies (percentages), means with standard deviations, and medians with an interquartile range. To control the effect of confounding variables when T2DM patients and non-T2DM patients were compared, we matched the cohorts using PSM. Multivariable logistic regression models were used to identify which study variables independently affected the IHM among men and women with HF and T2DM. Also, this multivariable method was applied for sensitivity analyses to confirm the results of the PSM. Results: A total of 28,894 patients were included. T2DM was present in 39.59%. Women with T2DM more frequently had atrial fibrillation, valvular heart disease, anemia, dementia, depression, and hyponatremia than men with T2DM. However, men had more coronary heart disease, chronic renal disease, COPD, and obstructive sleep apnea. All the procedures were significantly more commonly used among men than women. Blood transfusion was the only procedure more frequently identified among women with T2DM. For the sensitivity analysis in patients with T2DM hospitalized with HFrEF, we confirmed the results of the PSM, finding that women had a 14% higher risk of dying in the hospital than men (OR 1.14; 95% CI 1.01–1.35). Obesity seemed to have a protective effect (OR 0.85; 95% CI 0.73–0.98) on the in-hospital morality. Conclusions: Subjects with diabetes are admitted for HFrEF and have a greater number of comorbidities than non-diabetics. Diabetic women have a higher mortality rate than men with diabetes and all the procedures evaluated were significantly more often used among men than women.

## 1. Introduction

Heart failure (HF) is a prevalent disease primarily affecting elderly individuals [1,2]. The prognosis of heart failure is related to sex, age, etiology, left ventricular ejection fraction (EF), and comorbidities. In this group of patients, comorbid conditions are prevalent and will continue to increase as the result of the aging population. Comorbidities are already known to play a key role in heart failure [3]. Among comorbidities, diabetes mellitus and, particularly, type 2 diabetes mellitus (T2DM) are associated with an increased risk of cardiovascular disease [4]. Its prevalence is also increasing due to increasing rates of obesity in the general population [5].

Diabetes has been reported in up to 30% of patients with heart failure [6]. These are two entities with an increase in worldwide prevalence and chronic phenotype. It has been described that approximately 6% of newly diagnosed diabetic patients will develop heart failure during their life [7]. Diabetic patients may develop a so-called diabetic cardiomyopathy [8], and there is also evidence regarding abnormal left ventricular remodeling in these patients after the occurrence of acute myocardial infarction [9].

It is interesting that men predominate in most of the clinical studies conducted in heart failure patients; however, women are most often encountered in daily clinical practice [10]. This is important as women with heart failure are considered to have a better chance for survival, even though data about prognosis in patients with heart failure according to sex are controversial [11,12]. Previous studies have reported that women with heart failure are older than men and present with more hypertension or diabetes and less ischemic heart failure [12].

In addition, women are prone to develop less ventricular remodeling, right ventricular function is preserved, and they seem to be better protected against ventricular arrhythmias compared to men [13]. Interestingly, evidence-based therapies are less prescribed in women with heart failure, and they have a reduced ejection fraction compared to men [12]. Diabetes is an independent risk factor for mortality in patients with HF. In patients with heart failure and reduced ejection fraction, the presence of diabetes did not confer a protective effect on prognosis [12].

The objectives of this investigation were (1) to describe and compare the clinical characteristics and the use of diagnostic and therapeutic procedures among subjects hospitalized with heart failure and reduced ejection fraction (HFrEF) according to the presence of type 2 diabetes mellitus (T2DM) and sex; (2) using propensity score matching (PSM), we aimed to assess the effect of T2DM and sex on hospital outcomes among patients hospitalized with HFrEF; and (3) to identify which clinical variables were associated to in-hospital mortality (IHM) among patients hospitalized with HFrEF and T2DM according to sex.

## 2. Method

We conducted a retrospective cohort study based on administrative data. The database used is the Spanish National Hospital Discharge Database (SNHDD). In Spain, all hospitals are required by law to send records to the Ministry of Health Information on all their hospital discharges done in the previous month. The information contained in the SNHDD includes age, sex, up to 20 diagnoses (present at the time of admission or detected during the hospitalization), up to 20 procedures conducted during the hospital stay, duration of the hospitalization, and reason for discharge. The diagnosis and procedures are codified with the International Classification of Disease 10th version (ICD10).

In our investigation, we used the SNHDD for the years 2016, 2017, 2018, and 2019. Our study population is made up of subjects aged 40 and over with a primary diagnosis of HFrEF. The codes used to identify these patients are shown in Supplementary Table S1.

We defined as the exposed cohort those patients with a diagnosis of T2DM in any diagnosis position. The unexposed cohort were all those patients without a code for T2DM. Subjects who had a code for type 1 diabetes mellitus in any diagnosis field were excluded from the study population. Also, those with unspecified sex and missing data for age or duration of hospitalization were excluded.

Our main outcome variable was the IHM. All analyses were conducted separately for men and women and sex-differences described. The variables of interest were age, cardiovascular risk factors, clinical conditions, and diagnostic and therapeutic procedures. The cardiovascular risk factors included high blood pressure, current tobacco use, lipid metabolism disorders, and obesity. The clinical conditions analyzed were those of the Charlson Comorbidity Index (CCI) and were identified using the algorithms proposed by Sundararajan et al. for ICD10 codes in administrative databases [14]. Also, we analyzed the presence of atrial fibrillation, valvular heart disease, obstructive sleep apnea, dementia, depression, amyloidosis, hyponatremia, and hyperkalemia. The procedures studied were mechanical ventilation, vasopressor medication, heart echocardiogram, Mitra-clip, trans-catheter aortic valve implantation (TAVI), electrical cardioversion, dialysis, and red cell transfusion. Finally, we analyzed if patients had undergone “coronary artery bypass surgery” (including procedures where a blood vessel from another part of the body, usually the chest, leg, or arm, is extracted and attached to the coronary artery above and below the narrowed area or blockage) and “coronary artery dilatation with an intraluminal device (CADID)” (including procedures where dilation of one or more coronary arteries was conducted using an intraluminal device).

## 3. Statistical Methods

The descriptive statistics shown are the total and relative frequencies (percentages), means with standard deviations, and medians with an interquartile range. To compare percentages, a chi-square test was applied for the means and medians—we used a Students *t*-test and Mann–Whitney test, respectively.

To control the effect of the confounding variables when the exposed (T2DM patients) and unexposed (non-T2DM patients) groups were compared, we matched the cohorts using PSM. This method attempts to make study subpopulations more comparable across all the observed baseline covariates [15].

The variables initially included in the PSM model were age, cardiovascular risk factors, and all the clinical conditions analyzed. For PSM, we used the PSMATCH2 Stata module. The matching method chosen was one-to-one using calipers with a width equal to 0.2 of the standard deviation of the logit of the propensity score (PS) [15]. Using this method, we matched each T2DM man and women with a non-T2DM man and woman and a T2DM man with a T2DM woman. To assess the quality of the samples after PSM, we estimated the absolute standardized difference before and after matching. We conducted different logistic regression models to estimate the PS until we identified those variables that made our subpopulations comparable for the most relevant study variables (age and number of conditions included in the CCI). If any other variables could be added to the model that wouldn’t affect the quality of matching for these two variables, we maintained them in the PS.

Supplementary Table S2 shows the absolute standardized differences before and after PSM for the matching variables that were ultimately included. As can be seen in this table, none of the absolute standardized differences after PSM were above 10%, which would indicate a noteworthy imbalance [15].

The multivariable logistic regression models were used to identify which study variables independently affected the IHM among men and women with HFrEF and T2DM. Also, this multivariable method was applied for sensitivity analyses to confirm the results of the PSM. To do so, we analyzed the entire study population with HFrEF and T2DM to assess the effect of sex. Possible two-way interactions were examined. Model construction was done following the recommendations of Hosmer et al. [16]. Stata was the software used for descriptive, PSM, and analytical statistical analysis.

## 4. Results

The total number of individuals in our study population was 28,894. The overall prevalence of T2DM was 39.59% and the corresponding figures for men and women were 41.08% and 37.13%, respectively (*p* < 0.001).

Shown in Table 1 are the demographic and clinical characteristics of the study population according to sex and the presence of T2DM. Men with T2DM who were hospitalized with HFrEF from 2016 to 2019 were slightly, but significantly, younger than those without diabetes (73.18 years vs. 73.75 years; *p* = 0.002) and had a higher mean number of conditions included in the CCI (1.41 vs. 1.15: *p* < 0.001). As reported for men, women with T2DM were younger than those without this condition (79.02 years vs. 80.13 years; *p* < 0.001) and had a higher mean CCI (1 vs. 0.77; *p* < 0.001). Men with T2DM were significantly younger and had higher mean CCI than T2DM women (*p* < 0.001).

Regarding the cardiovascular risk factors, the prevalence was higher among T2DM men and women than non-T2DM men and women for high blood pressure, lipid metabolism disorders, and obesity, whereas current tobacco use was more frequent among non-T2DM men and women (all *p* < 0.05). When men and women with T2DM were compared, we observed significantly higher values for high blood pressure and obesity among women and tobacco use and lipid disorders among men (Table 1).

Of the clinical conditions described, a significantly higher coding was found for both men and women with T2DM than for men and women without diabetes or coronary heart disease, chronic renal disease, chronic obstructive respiratory disease (COPD), anemia, obstructive sleep apnea, and hyperkalemia. Atrial fibrillation was only more prevalent for T2DM men. On the other hand, men without T2DM had more valvular heart disease, dementia, and amyloidosis than T2DM men. Among women, only valvular heart disease was more frequent among those not suffering from T2DM.

The prevalence of all the clinical conditions analyzed, with the exception of amyloidosis and hyperkalemia, showed sex-differences among the T2DM patients (Table 1). Women with T2DM more frequently had a code recorded for atrial fibrillation, valvular heart disease, anemia, dementia, depression, and hyponatremia than men with T2DM. However, men had more coronary heart disease, chronic renal disease, COPD, and obstructive sleep apnea.

For the procedures investigated, men and women with T2DM underwent CADID and dialysis in a higher proportion than non-T2DM men and women. Only among women were red cell transfusions received more frequently than those without T2DM. However, non-T2DM men and women underwent electrical cardioversions during their hospitalization more frequently than men and women with T2DM.

Remarkable sex differences are found in the use of procedures among patients with T2DM. Almost all the procedures were significantly more frequently used among men than women (vasopressor medication, heart echocardiogram, CADID, electrical cardioversion, and dialysis). Red cell transfusion was the only procedure more frequently recorded for T2DM women.

The median LOHS was seven days for all the subgroups compared with no significant differences by T2DM status nor sex. The crude IHM was higher among men without than men with T2DM (8.72% vs. 7.17%; *p* < 0.001). The same was observed for women (12.42% women without T2DM vs. 10.7% for T2DM women; *p* < 0.001). Women with T2DM died in the hospital after being hospitalized for HFrEF in a higher proportion than men with T2DM (10.7% vs. 7.17%; *p* < 0.001)

The distribution according to the study variables after the PSM for men with and without T2DM who were hospitalized with SHF HFrEF are shown in Table 2.

The number of men with HFrEF matched was 7381. After PSM, the differences in the mean age and CCI became insignificant and the distance in the prevalence of clinical conditions was reduced. The proportion of T2DM men who underwent CADID remained significantly higher than for those without T2DM (2.38% vs. 1.77%; *p* = 0.009). No significant differences for any other procedure were found. After PSM, the IHM among men without T2DM was 8.32%, compared with 7.17% for those with T2DM (*p* = 0.009)

The subpopulations of women with and without T2DM who were hospitalized with HFrEF obtained using PSM are shown in Table 3. After matching the mean age and CCI, the groups were not significantly different, and the rest of the clinical variables showed closer values. The proportion of T2DM women who received red cell transfusions were higher than for non-T2DM, whereas the use of electrical cardioversion was lower. Once matching was done, PSM women without T2DM still showed a higher IHM than for those with this disease (12.26% vs. 10.70%; *p* = 0.031).

The comparison of men and women with T2DM who were hospitalized for HFREF after PSM is shown in Table 4.

As can be seen in Table 5, the distribution according to age and CCI was similar after PSM. However, most clinical conditions, with the exception of high blood pressure, maintained the differences described before PSM (Table 1). Regarding the procedures, after matching the heart echocardiograms, CADID and electrical cardioversion were more commonly used among men than women. Women with T2DM had a higher IHM than men with T2DM after PSM (10.7% vs. 8.8%; *p* = 0.004).

The results of the multivariable regression analysis to identify the variables that are independently associated with IHM among men and women with type 2 diabetes mellitus who were hospitalized with HFrEF are shown in Table 5.

For both sexes, the probability of dying in the hospital rose with age and among those with a diagnosis of dementia, hyponatremia, or hyperkalemia. Chronic renal disease was a risk factor only among women.

Those men and women with T2DM who required mechanical ventilation or dialysis during their hospitalization also had a higher IHM. Only among men with T2DM was there a need for vasopressor medication, electrical cardioversion, and red cell transfusion, which were also associated with a higher mortality.

When we analyzed the entire population of T2DM patients hospitalized with HFrEF in a sensitivity analysis, we confirmed the results of the PSM finding, which found that women had a 14% higher risk of dying in the hospital than men (OR 1.14; 95% CI 1.01–1.35). We also found that obesity seemed to have a protective effect (OR 0.85; 95% CI 0.73–0.98) on the IHM.

## 5. Discussion

The results of our research based on the ICD-10 classification show that, among hospitalizations for HFrEF, there are clinical and prognostic differences between patients with and without diabetes and also in relation to sex.

Diabetic subjects with HFrEF were younger but had a greater number of cardiovascular and non-cardiovascular comorbidities. The most common comorbid conditions were hypertension, dyslipidemia, obesity, ischemic heart disease, chronic renal failure, anemia, and obstructive sleep apnea. All these risk factors and associated pathologies were more prevalent in diabetic patients, have been described by other authors, and are associated with the natural evolution of diabetes [2,6,7].

On the other hand, the presence of dementia, AF, amyloidosis, and valvular heart disease was more prevalent in non-diabetic patients than in patients with diabetes. This may be due to a higher prevalence of these chronic diseases associated with the advanced age of non-diabetic patients with HFrEF [2].

Subjects with diabetes more frequently received invasive procedures for disease management such as CADID, dialysis, and red blood cell transfusion. These findings are congruent since patients with diabetes tend to have a higher incidence of diffuse atherosclerotic coronary artery disease, which often leads to the need for coronary by-pass procedures [5,17]. On the other hand, diabetes is the most frequent risk factor for entering dialysis in our country. The higher frequency of renal dysfunction and hyperkalemia recorded in hospitalizations of patients with diabetes HFrEF justifies the use of invasive procedures [18]. Recent advances with new potassium binding treatments may help avoid these procedures in patients with hyperkalemia and cardiorenal syndrome [19].

Subjects with heart failure and reduced ejection fraction had a greater degree of anemia that required invasive procedures. In this sense, it seems of interest to us to evaluate through further prospective studies the safety of transfusion in the treatment of anemia in diabetes and cardiorenal syndrome. There are therapies that improve the prognosis of HF and anemia with iron deficiency, such as the use of iron carboxymaltose, as has been demonstrated in the AFFIRM trial [20].

After adjusting with PSM in relation to sex between diabetics and non-diabetic patients, it was observed that younger male subjects under 50 years of age and male patients between 65 and 74 years of age with diabetes had a higher rate of hospitalization for HF than non-diabetic male subjects. In relation to diabetic and non-diabetic women, the same differences were observed for the same age groups. These findings are related to the fact that diabetes is a risk factor that increases the risk of hospitalization for HF by approximately five times [17]. Furthermore, the presence of ischemic heart disease in diabetic patients is greater and can appear at younger ages and increase the risk of hospitalization for HFrEF [5,6,17].

When we performed PSM among diabetic subjects by sex, we observed a higher frequency of women over 85 years of age during hospital admission. These findings may be due to the greater life expectancy of women than men and to the greater frequency of atrial fibrillation, chronic renal failure, and obesity, factors that increase the risk of developing heart failure over the years [21]. The presence of hyponatremia was greater in diabetic women than in diabetic men in the study. This indicates a higher risk of mortality due to this electrolyte alteration [22,23,24].

Women hospitalized for diabetes in our setting had a lower rate of prior echocardiograms than men. Some studies relate that women undergo fewer complementary tests than men for the management of heart failure or atrial fibrillation [21]. However, on the other hand, they received a greater number of noninvasive mechanical ventilations, probably due to a worse cardiorespiratory situation than men upon admission. In addition, they were transfused more often than men, indicating a more severe degree of anemia than men with diabetes.

In relation to the in-hospital mortality observed in the series analyzed, we found a lower rate for patients with HF and reduced EF with diabetes compared to non-diabetics, and in contrast to other studies [25]. These findings have been reported in other studies where diabetic subjects have lower in-hospital mortality than non-diabetics. Numerous factors have been attributed to this observation, such as the younger age of patients admitted for diabetes, which is the factor most associated with in-hospital mortality, or the obesity paradox observed in some patients with diabetes during admission [26].

However, when we compare in-hospital mortality between women and men with diabetes, the former seems to have a higher in-hospital mortality. Chin-Hsiao et al. reported a higher in-hospital mortality in men [27]. This result may be explained by the higher number of women over 85 years of age who are hospitalized and the higher disease burden in terms of anemic syndrome, chronic renal dysfunction, dementia, and depression that probably reflect a higher degree of advanced and terminal heart failure than male patients without diabetes [21].

To our knowledge, this is the first analysis of the clinical characteristics and hospital outcomes among patients hospitalized with HFrEF according to the presence of T2DM that assessed the sex differences in a national population-based database. The major strengths of this study include: the number of subjects, the long surveillance period (4 years), the very high territory-wide population coverage (>95% of all hospital admissions), the ability to analyze many diagnostic and therapeutic procedures conducted during the hospitalization, and that the PSM was conducted to control for the differences in the demographic and baseline clinical characteristics between the study subpopulations. Another strength is that the use of the ICD-10 coding allows us to reliably identify subjects with HFrEF and T2DM [28,29].

As for the limitations of our study, since it is a broad clinical–administrative study of all the Spanish hospitals, we do not have important data such as NYHA functional class, specific left ventricular ejection fraction, NT-proBNP levels, time of disease evolution, functional status of patients, glycated hemoglobin levels, or pharmacological treatment received for HFrEF (beta-blockers, valsartan sacubitril, aldosterone antagonists, or treatment with SGLT2 inhibitors). The follow-up time in this study, due to its characteristics, is reduced to hospitalization, so we do not know the readmission and out-of-hospital mortality rates of patients with and without diabetes and HFrEF.

In conclusion, subjects with diabetes that are admitted for HFrEF are relatively younger and have a greater number of comorbidities than non-diabetics. Diabetic women have a higher mortality rate than men with diabetes, and all the procedures evaluated were used significantly more among men than women.

## Figures and Tables

**Table 1:** Demographic and clinical characteristics of subjects hospitalized with systolic heart failure in Spain from 2016 to 2019 according to sex and the presence of type 2 diabetes mellitus.

	MEN			WOMEN			BOTH SEXES		
	TDM2	NoTDM2	*p*	TDM2	NoTDM2	*p*	TDM2	NoTDM2	*p*
Age, mean (SD) *	73.18 (10.72)	73.75 (13.09)	0.002	79.02 (9.44)	80.13 (11.34)	<0.001	75.25 (10.66)	76.26 (12.81)	<0.001
CCI, mean (SD) *	1.41 (1.05)	1.15 (1.02)	<0.001	1 (0.87)	0.77 (0.84)	<0.001	1.26 (1.01)	1 (0.97)	<0.001
High blood pressure, *n* (%) *	2930 (39.7)	3186 (30.09)	<0.001	1869 (46.07)	2590 (37.71)	<0.001	4799 (41.96)	5776 (33.09)	<0.001
Current tobacco use, *n* (%) *	922 (12.49)	1475 (13.93)	0.005	117 (2.88)	281 (4.09)	0.001	1039 (9.08)	1756 (10.06)	0.006
Lipid metabolism disorders, *n* (%) *	3841 (52.04)	3147 (29.73)	<0.001	1882 (46.39)	1914 (27.86)	<0.001	5723 (50.03)	5061 (28.99)	<0.001
Obesity, *n* (%) *	1237 (16.76)	959 (9.06)	<0.001	932 (22.97)	834 (12.14)	<0.001	2169 (18.96)	1793 (10.27)	<0.001
Coronary heart disease, *n* (%) *	3870 (52.43)	3957 (37.38)	<0.001	1256 (30.96)	1318 (19.19)	<0.001	5126 (44.82)	5275 (30.22)	<0.001
Chronic renal disease, *n* (%) *	3230 (43.76)	3188 (30.11)	<0.001	1699 (41.88)	1752 (25.51)	<0.001	4929 (43.09)	4940 (28.3)	<0.001
COPD, *n* (%) *	1544 (20.92)	1904 (17.98)	<0.001	240 (5.92)	325 (4.73)	0.007	1784 (15.6)	2229 (12.77)	<0.001
Atrial fibrillation, *n* (%) *	3250 (44.03)	5051 (47.71)	<0.001	2013 (49.62)	3461 (50.39)	0.438	5263 (46.01)	8512 (48.76)	<0.001
Valvular heart disease, *n* (%) *	2553 (34.59)	4031 (38.07)	<0.001	1650(40.67)	2942 (42.83)	0.027	4203 (36.75)	6973 (39.95)	<0.001
Anemia, *n* (%) *	877 (11.88)	914 (8.63)	<0.001	651 (16.05)	766 (11.15)	<0.001	1528 (13.36)	1680 (9.62)	<0.001
Obstructive sleep apnea, *n* (%) *	741 (10.04)	690 (6.52)	<0.001	252 (6.21)	190 (2.77)	<0.001	993 (8.68)	880 (5.04)	<0.001
Dementia, *n* (%) *	128 (1.73)	242 (2.29)	0.010	182 (4.49)	310 (4.51)	0.948	310 (2.71)	552 (3.16)	0.027
Depression, *n* (%) *	169 (2.29)	248 (2.34)	0.817	295 (7.27)	489 (7.12)	0.765	464 (4.06)	737 (4.22)	0.491
Amyloidosis, *n* (%)	38 (0.51)	112 (1.06)	<0.001	16 (0.39)	25 (0.36)	0.802	54 (0.47)	137 (0.78)	0.001
Hyponatremia, *n* (%) *	161 (2.18)	254 (2.4)	0.339	147 (3.62)	226 (3.29)	0.354	308 (2.69)	480 (2.75)	0.771
Hyperkalemia, *n* (%)	163 (2.21)	163 (1.54)	0.001	90 (2.22)	93 (1.35)	0.001	253 (2.21)	256 (1.47)	<0.001
Mechanical ventilation, *n* (%)	284 (3.85)	379 (3.58)	0.349	173 (4.26)	235 (3.42)	0.025	457 (4)	614 (3.52)	0.035
Vasopressors medication, *n* (%) *	84 (1.14)	141 (1.33)	0.251	17 (0.42)	37 (0.54)	0.389	101 (0.88)	178 (1.02)	0.245
Heart echocardiogram, *n* (%) *	2387 (32.34)	3432 (32.42)	0.913	1024 (25.24)	1757 (25.58)	0.695	3411 (29.82)	5189 (29.73)	0.862
Coronary artery bypass surgery, *n* (%)	12 (0.16)	28 (0.26)	0.154	3 (0.07)	6 (0.09)	0.813	15 (0.13)	34 (0.19)	0.199
CADID, *n* (%) *	176 (2.38)	176 (1.66)	0.001	52 (1.28)	51 (0.74)	0.005	228 (1.99)	227 (1.3)	<0.001
Mitra-clip, *n* (%)	4 (0.05)	9 (0.09)	0.450	0 (0)	2 (0.03)	0.277	4 (0.03)	11 (0.06)	0.306
TAVI, *n* (%)	8 (0.11)	8 (0.08)	0.468	3 (0.07)	13 (0.19)	0.128	11 (0.1)	21 (0.12)	0.546
Electrical cardioversion, *n* (%) *	116 (1.57)	227 (2.14)	0.006	27 (0.67)	84 (1.22)	0.005	143 (1.25)	311 (1.78)	<0.001
Dialysis, *n* (%) *	95 (1.29)	96 (0.91)	0.014	34 (0.84)	29 (0.42)	0.006	129 (1.13)	125 (0.72)	<0.001
Red cell transfusion, *n* (%) *	214 (2.9)	276 (2.61)	0.236	147 (3.62)	126 (1.83)	<0.001	361 (3.16)	402 (2.3)	<0.001
LOHS, median (IQR)	7 (8)	7 (7)	0.775	7 (8)	7 (7)	0.063	7 (8)	7 (7)	0.081
In hospital mortality, *n* (%) *	529 (7.17)	923 (8.72)	<0.001	434 (10.7)	853 (12.42)	0.007	963 (8.42)	1776 (10.17)	<0.001

* Significant difference (*p* < 0.05) between men with T2DM and women with T2DM. CCI; Charlson Comorbidity index. T2DM; type 2 diabetes mellitus. COPD; Chronic obstructive respiratory disease. CADID; Coronary artery dilatation with intraluminal device. TAVI; Trans-catheter aortic valve implantation. LOHS; Length of hospital stay.

**Table 2:** Demographic and clinical characteristics, after propensity score matching, of men with and without type 2 diabetes mellitus hospitalized with systolic heart failure in Spain from 2016 to 2019.

	T2DM (*n* = 7381)	No T2DM (*n* = 7381)	*p* Value
Age, mean (SD)	73.18 (10.72)	73.23 (11.51)	0.799
40–54 years, *n* (%)	393 (5.32)	528 (7.15)	<0.001
55–64 years, *n* (%)	1223 (16.57)	1140 (15.45)	0.062
65–74 years, *n* (%)	2170 (29.4)	1937 (26.24)	<0.001
75–84 years, *n* (%)	2512 (34.03)	2562 (34.71)	0.386
85+ years, *n* (%)	1083 (14.67)	1214 (16.45)	0.003
CCI, mean (SD)	1.41 (1.05)	1.4 (1.05)	0.645
High blood pressure, *n* (%)	2930 (39.7)	2252 (30.51)	<0.001
Current tobacco use, *n* (%)	922 (12.49)	1027 (13.91)	0.011
Lipid metabolism disorders, *n* (%)	3841 (52.04)	2381 (32.26)	<0.001
Obesity, *n* (%)	1237 (16.76)	705 (9.55)	<0.001
Coronary heart disease, *n* (%)	3870 (52.43)	3153 (42.72)	<0.001
Chronic renal disease, *n* (%)	3230 (43.76)	2582 (34.98)	<0.001
COPD, *n* (%)	1544 (20.92)	1588 (21.51)	0.376
Atrial fibrillation, *n* (%)	3250 (44.03)	3515 (47.62)	<0.001
Valvular heart disease, *n* (%)	2553 (34.59)	2812 (38.1)	<0.001
Anemia, *n* (%)	877 (11.88)	645 (8.74)	<0.001
Obstructive sleep apnea, *n* (%)	741 (10.04)	542 (7.34)	<0.001
Dementia, *n* (%)	128 (1.73)	183 (2.48)	0.002
Depression, *n* (%)	169 (2.29)	177 (2.4)	0.663
Amyloidosis, *n* (%)	38 (0.51)	63 (0.85)	0.013
Hyponatremia, *n* (%)	161 (2.18)	162 (2.19)	0.955
Hyperkalemia, *n* (%)	163 (2.21)	103 (1.4)	<0.001
Mechanical ventilation, *n* (%)	284 (3.85)	267 (3.62)	0.460
Vasopressors medication, *n* (%)	84 (1.14)	105 (1.42)	0.124
Heart echocardiogram, *n* (%)	2387 (32.34)	2345 (31.77)	0.480
Coronary artery bypass surgery, *n* (%)	12 (0.16)	21 (0.28)	0.117
CADID, *n* (%)	176 (2.38)	131 (1.77)	0.009
Mitra-clip, *n* (%)	4 (0.05)	7 (0.09)	0.366
TAVI, *n* (%)	8 (0.11)	6 (0.08)	0.593
Electrical cardioversion, *n* (%)	116 (1.57)	143 (1.94)	0.091
Dialysis, *n* (%)	95 (1.29)	82 (1.11)	0.326
Red cell transfusion, *n* (%)	214 (2.9)	195 (2.64)	0.341
LOHS, median (IQR)	7 (8)	7 (8)	0.224
In hospital mortality, *n* (%)	529 (7.17)	614 (8.32)	0.009

T2DM; type 2 diabetes mellitus. CCI; Charlson Comorbidity index. COPD; Chronic obstructive respiratory disease. CADID; Coronary Artery dilatation with intraluminal device. TAVI; Trans-catheter aortic valve implantation. LOHS; Length of hospital stay.

**Table 3:** Demographic and clinical characteristics, after propensity score matching, of women with and without type 2 diabetes mellitus hospitalized with systolic heart failure in Spain from 2016 to 2019.

	T2DM (*n* = 4047)	No T2DM (*n* = 4047)	*p* Value
Age, mean (SD)	79.02 (9.44)	78.79 (10.37)	0.296
40–54 years, *n* (%)	62 (1.53)	112 (2.76)	<0.001
55–64 years, *n* (%)	266 (6.56)	300 (7.39)	0.138
65–74 years, *n* (%)	825 (20.34)	738 (18.19)	0.014
75–84 years, *n* (%)	1634 (40.28)	1595 (39.31)	0.376
85+ years, *n* (%)	1270 (31.3)	1312 (32.34)	0.317
CCI, mean (SD)	1 (0.87)	1 (0.87)	0.980
High blood pressure, *n* (%)	1869 (46.07)	1535 (37.84)	<0.001
Current tobacco use, *n* (%)	117 (2.88)	176 (4.34)	<0.001
Lipid metabolism disorders, *n* (%)	1882 (46.39)	1196 (29.48)	<0.001
Obesity, *n* (%)	932 (22.97)	526 (12.97)	<0.001
Coronary heart disease, *n* (%)	1256 (30.96)	905 (22.31)	<0.001
Chronic renal disease, *n* (%)	1699 (41.88)	1252 (30.86)	<0.001
COPD, *n* (%)	240 (5.92)	268 (6.61)	0.199
Atrial fibrillation, *n* (%)	2013 (49.62)	2055 (50.65)	0.351
Valvular heart disease, *n* (%)	1650 (40.67)	1758 (43.33)	0.015
Anemia, *n* (%)	651 (16.05)	460 (11.34)	<0.001
Obstructive sleep apnea, *n* (%)	252 (6.21)	136 (3.35)	<0.001
Dementia, *n* (%)	182 (4.49)	228 (5.62)	0.020
Depression, *n* (%)	295 (7.27)	319 (7.86)	0.314
Amyloidosis, *n* (%)	16 (0.39)	16 (0.39)	0.999
Hyponatremia, *n* (%)	147 (3.62)	126 (3.11)	0.196
Hyperkalemia, *n* (%)	90 (2.22)	59 (1.45)	0.010
Mechanical ventilation, *n* (%)	173 (4.26)	149 (3.68)	0.172
Vasopressor medication, *n* (%)	17 (0.42)	26 (0.64)	0.169
Heart echocardiogram, *n* (%)	1024 (25.24)	1009 (24.87)	0.701
Coronary artery bypass surgery, *n* (%)	3 (0.07)	5 (0.12)	0.479
CADID, *n* (%)	52 (1.28)	35 (0.86)	0.067
Mitra-clip, *n* (%)	0 (0)	1 (0.02)	0.317
TAVI, *n* (%)	3 (0.07)	9 (0.22)	0.083
Electrical cardioversion, *n* (%)	27 (0.67)	50 (1.23)	0.008
Dialysis, *n* (%)	34 (0.84)	28 (0.69)	0.444
Red cell transfusion, *n* (%)	147 (3.62)	86 (2.12)	<0.001
LOHS, median (IQR)	7 (8)	7 (7)	0.454
In hospital mortality, *n* (%)	434 (10.70)	496 (12.26)	0.031

T2DM; type 2 diabetes mellitus. CCI Charlson Comorbidity index. COPD; Chronic obstructive respiratory disease. CADID; Coronary Artery dilatation with intraluminal device TAVI; Trans-catheter aortic valve implantation. LOHS; Length of hospital stay.

**Table 4:** Demographic and clinical characteristics, after propensity score matching, of men and women with type 2 diabetes mellitus hospitalized with systolic heart failure in Spain from 2016 to 2019.

	T2DM WOMEN (*n* = 4047)	T2DM MEN (*n* = 4047)	*p* Value
Age, mean (SD)	79.02 (9.44)	78.76 (8.97)	0.204
40–54 years, *n* (%)	62 (1.53)	60 (1.48)	0.850
55–64 years, *n* (%)	266 (6.56)	267 (6.58)	0.964
65–74 years, *n* (%)	825 (20.34)	932 (22.97)	0.004
75–84 years, *n* (%)	1634 (40.28)	1774 (43.73)	0.002
85+ years, *n* (%)	1270 (31.3)	1024 (25.24)	<0.001
CCI, mean (SD)	1.03 (0.87)	1.06 (0.86)	0.119
High blood pressure, *n* (%)	1869 (46.07)	1805 (44.49)	0.153
Current tobacco use, *n* (%)	117 (2.88)	339 (8.36)	<0.001
Lipid metabolism disorders, *n* (%)	1882 (46.39)	2011 (49.57)	0.004
Obesity, *n* (%)	932 (22.97)	521 (12.84)	<0.001
Coronary heart disease, *n* (%)	1256 (30.96)	1894 (46.68)	<0.001
Chronic renal disease, *n* (%)	1699 (41.88)	1585 (39.07)	0.010
COPD, *n* (%)	240 (5.92)	680 (16.76)	<0.001
Atrial fibrillation, *n* (%)	2013 (49.62)	1903 (46.91)	0.015
Valvular heart disease, *n* (%)	1650 (40.67)	1429 (35.22)	<0.001
Anemia, *n* (%)	651 (16.05)	475 (11.71)	<0.001
Obstructive sleep apnea, *n* (%)	252 (6.21)	301 (7.42)	0.031
Dementia, *n* (%)	182 (4.49)	75 (1.85)	<0.001
Depression, *n* (%)	295 (7.27)	99 (2.44)	<0.001
Amyloidosis, *n* (%)	16 (0.39)	31 (0.76)	0.028
Hyponatremia, *n* (%)	147 (3.62)	94 (2.32)	0.001
Hyperkalemia, *n* (%)	90 (2.22)	94 (2.32)	0.765
Mechanical ventilation, *n* (%)	173 (4.26)	119 (2.93)	0.001
Vasopressors medication, *n* (%)	17 (0.42)	26 (0.64)	0.169
Heart echocardiogram, *n* (%)	1024 (25.24)	1134 (27.95)	0.006
Coronary artery bypass surgery, *n* (%)	3 (0.07)	2 (0.05)	0.655
CADID, *n* (%)	52 (1.28)	83 (2.05)	0.007
Mitra-clip, *n* (%)	0 (0)	1 (0.02)	0.317
TAVI, *n* (%)	3 (0.07)	6 (0.15)	0.317
Electrical cardioversion, *n* (%)	27 (0.67)	50 (1.23)	0.008
Dialysis, *n* (%)	34 (0.84)	45 (1.11)	0.214
Red cell transfusion, *n* (%)	147 (3.62)	98 (2.42)	0.001
LOHS, median (IQR)	7 (8)	7 (7)	0.295
In hospital mortality, *n* (%)	434 (10.7)	357 (8.8)	0.004

T2DM; type 2 diabetes mellitus. CCI Charlson Comorbidity index. COPD; Chronic obstructive respiratory disease. CADID; Coronary Artery dilatation with intraluminal device. TAVI; Trans-catheter aortic valve implantation. LOHS; Length of hospital stay.

**Table 5:** Variables independently associated with in-hospital mortality among men and women with type 2 diabetes mellitus hospitalized with systolic heart failure in Spain from 2016 to 2019.

	Men with T2DM	Women with T2DM	Both Sexes with T2DM
40–54 years	Reference	Reference	Reference
55–64 years	1.54 (0.7–3.41)	1.14 (0.41–3.16)	1.32 (0.69–2.54)
65–74 years	1.81 (0.84–3.89)	1.29 (0.49–3.37)	1.64 (0.88–3.06)
75–84 years	4.89 (2.31–10.38)	2.86 (1.11–7.35)	3.78 (2.05–6.96)
85+ years	7.58 (3.52–16.3)	4.67 (1.81–12.08)	6.2 (3.34–11.5)
Obesity	NS	NS	0.85 (0.73–0.98)
Chronic renal disease	NS	1.46 (1.15–1.85)	1.29 (1.1–1.51)
Dementia	1.77 (1.08–2.91)	2.01 (1.36–2.96)	1.93 (1.43–2.62)
Hyponatremia	2.78 (1.79–4.32)	2.21 (1.43–3.41)	2.48 (1.83–3.37)
Hyperkalemia	2.42 (1.56–3.76)	2.31 (1.39–3.85)	2.41 (1.73–3.35)
Mechanical ventilation	5.92 (4.23–8.29)	3.57 (2.41–5.27)	4.68 (3.64–6.03)
Vasopressors medication	3.21 (1.72–5.96)	NS	2.81 (1.61–4.91)
Electrical cardioversion	2.1 (1.07–4.1)	NS	2.02 (1.13–3.6)
Red cell transfusion	1.67 (1.07–2.61)	NS	NS
Dialysis	5.64 (3.37–9.45)	4.25 (1.84–9.8)	5.36 (3.49–8.25)
Female	NA	NA	1.14 (1.01–1.35)

T2DM; type 2 diabetes mellitus. NS; not significant. NA; Not adequate. No significant interactions were found in any of these models.

## Data Availability

Not applicable.

## Supplementary Materials

The following supporting information can be downloaded at: https://www.mdpi.com/article/10.3390/jcm11041030/s1, Table S1: ICD10 codes for clinical conditions, laboratory results and procedures analyzed in the study; Table S2: Absolute standardized differences before and after Propensity Score Matching (PSM) for men and women with T2DM matched with men and women without T2DM and for T2DM men matched with T2DM women hospitalized with heart failure with reduced ejection fraction in Spain (2016–2019).

## References

[1] Emmons-Bell S., Johnson C., Roth G. (2022). Prevalence, incidence and survival of heart failure: A systematic review. Heart.

[2] Groenewegen A., Rutten F.H., Mosterd A., Hoes A.W. (2020). Epidemiology of heart failure. Eur. J. Heart Fail..

[3] Shah S.J., Gheorghiade M. (2008). Heart failure with preserved ejection fraction: Treat now by treating comorbidities. JAMA.

[4] Masarone D., Pacileo R., Pacileo G. (2021). Use of disease-modifying drugs in diabetic patients with heart failure with reduced ejection fraction. Heart Fail. Rev..

[5] DeFronzo R.A., Ferrannini E., Groop L., Henry R.R., Herman W.H., Holst J.J., Hu F.B., Kahn C.R., Raz I., Shulman G.I. (2015). Type 2 diabetes mellitus. Nat. Rev. Dis. Primers.

[6] Lehrke M., Marx N. (2017). Diabetes Mellitus and Heart Failure. Am. J. Med..

[7] Wilkinson M.J., Zadourian A., Taub P.R. (2019). Heart failure and diabetes mellitus: Defining the problem and exploring the interrelationship. Am. J. Cardiol..

[8] Dillmann W.H. (2019). Diabetic Cardiomyopathy. Circ. Res..

[9] Tribouilloy C., Rusinaru D., Mahjoub H., Souliere V., Levy F., Peltier M., Slama M., Massy Z. (2008). Prognosis of heart failure with preserved ejection fraction: A 5 year prospective population-based study. Eur. Heart J..

[10] Martınez-Selles M., Garcıa Robles J.A., Prieto L., Dominguez Munos M., Frades E., Dıaz-Castro O., Almendral J. (2003). Systolic dysfunction is a predictor of long term mortality in men but not in women with heart failure. Eur. Heart J..

[11] Nicol E.D., Fittall B., Roughton M., Cleland J.G., Dargie H., Cowie M.R. (2008). NHS heart failure survey: A survey of acute heart failure admissions in England, Wales and Northern Ireland. Heart.

[12] Martínez-Sellés M., Doughty R.N., Poppe K., Whalley G.A., Earle N., Tribouilloy C., McMurray J.J., Swedberg K., Køber L., Berry C. (2012). Meta-Analysis Global Group in Chronic Heart Failure (MAGGIC). Gender and survival in patients with heart failure: Interactions with diabetes and aetiology. Results from the MAGGIC individual patient meta-analysis. Eur. J. Heart Fail..

[13] Martınez-Selles M. (2007). What do women have in their hearts?. Rev. Esp. Cardiol..

[14] Sundararajan V., Henderson T., Perry C., Muggivan A., Quan H., Ghali W.A. (2004). New ICD-10 version of the Charlson comorbidity index predicted in-hospital mortality. J. Clin. Epidemiol..

[15] Austin P.C. (2011). Comparing paired vs non-paired statistical methods of analyses when making inferences about absolute risk reductions in propensity-score matched samples. Stat. Med..

[16] Hosmer D.W., Lemeshow S., Sturdivant R.X. (2013). Applied Logistic Regression.

[17] Zhou Y., Wang M., Wang S., Li N., Zhang S., Tang S., Shi Q., Zhao Y., Li J., Zeng Y. (2021). Diabetes in Patients with Heart Failure with Reduced Ejection Fraction During Hospitalization: A Retrospective Observational Study. Front. Endocrinol..

[18] Gregorich M., Heinzel A., Kammer M., Meiselbach H., Böger C., Eckardt K.U., Mayer G., Heinze G., Oberbauer R. (2021). A prediction model for the decline in renal function in people with type 2 diabetes mellitus: Study protocol. Diagn. Progn. Res..

[19] Sarwar C.M., Papadimitriou L., Pitt B., Piña I., Zannad F., Anker S.D., Gheorghiade M., Butler J. (2016). Hyperkalemia in Heart Failure. J. Am. Coll. Cardiol..

[20] McEwan P., Ponikowski P., Davis J.A., Rosano G., Coats A.J.S., Dorigotti F., O’Sullivan D., Ramirez de Arellano A., Jankowska E.A. (2021). Ferric carboxymaltose for the treatment of iron deficiency in heart failure: A multinational cost-effectiveness analysis utilising AFFIRM-AHF. Eur. J. Heart Fail..

[21] Swaraj S., Kozor R., Arnott C., Di Bartolo B.A., Figtree G.A. (2021). Heart Failure with Reduced Ejection Fraction-Does Sex Matter?. Curr. Heart Fail. Rep..

[22] Kanelidis A.J., Imamura T., Yang B., Miller T.A., Bharmal M., Kim G., Sayer G., Uriel N. (2021). The Clinical Importance of Hyponatremia in Patients with Left Ventricular Assist Devices. ASAIO J..

[23] Suwanto D., Dewi I.P., Fagi R.A. (2021). Hyponatremia in heart failure: Not just 135 to 145. J. Basic Clin. Physiol. Pharmacol..

[24] Su Y., Ma M., Zhang H., Pan X., Zhang X., Zhang F., Lv Y., Yan C. (2020). Prognostic value of serum hyponatremia for outcomes in patients with heart failure with preserved ejection fraction: An observational cohort study. Exp. Ther. Med..

[25] Fudim M., Devaraj S., Chukwurah M., Ajam T., Razaghizad A., Salah H.M., Sharma A., Savarese G., Vaduganathan M., Kamalesh M. (2022). Prognosis for patients with heart failure and reduced ejection fraction with and without diabetes: A 7 year nationwide veteran administration analysis. Int. J. Cardiol..

[26] Ohori K., Yano T., Katano S., Kouzu H., Honma S., Shimomura K., Inoue T., Takamura Y., Nagaoka R., Koyama M. (2021). High percent body fat mass predicts lower risk of cardiac events in patients with heart failure: An explanation of the obesity paradox. BMC Geriatr..

[27] Chin-Hsiao T. (2004). Mortality and Causes of Death in a National Sample of Diabetic Patients in Taiwan. Diabetes Care.

[28] Bosco-Lévy P., Duret S., Picard F., Dos Santos P., Puymirat E., Gilleron V., Blin P., Chatellier G., Looten V., Moore N. (2019). Diagnostic accuracy of the International Classification of Diseases, Tenth Revision, codes of heart failure in an administrative database. Pharmacoepidemiol. Drug Saf..

[29] Khokhar B., Jette N., Metcalfe A., Cunningham C.T., Quan H., Kaplan G.G., Butalia S., Rabi D. (2016). Systematic review of validated case definitions for diabetes in ICD-9-coded and ICD-10-coded data in adult populations. BMJ Open.

