# Supplementary material for: Clinical Characteristics, Management, and In-Hospital Mortality in Patients with Heart Failure with Reduced Ejection Fraction According to Sex and the Presence of Type 2 Diabetes Mellitus
Source: J Clin Med. 2022 Feb 16;11(4):1030. doi: 10.3390/jcm11041030 (PMC8878152; doi:10.3390/jcm11041030)
Supplement: Supplementary file 1 [file jcm-11-01030-s001.zip › jcm-1526491-supplementary.pdf]

Supplementary Table S1. ICD10 codes for clinical conditions, laboratory results and procedures analyzed in the study

**Condition/Laboratory results/Procedures**	**ICD 10 codes**
Systolic heart failure	I50.2; I50.20; I50.2;I50.22; I50.23; I50.4; I50.40; I50.41;I50.42; I50.43
Type 1 diabetes mellitus	E10.xxx
Type 2 diabetes mellitus	E11.xxx
High blood pressure	I10; I16.6
Current tobacco use	Z72.0; Z87.89; F17.2xxx
Lipid metabolism disorders	E78.x
Obesity	E66.x
Atrial fibrillation	I48.0; I48.1; I48.2; I48.91
Valvular heart disease	I05.x; I06.x; I07.x; I08.x; I34.x;I35.x; I36.x; I37.x
Anemia	D50.0; D50.8; D50.9; D51.x-D53.x
Obstructive sleep apnea	G47.33
Dementia	F01-F03
Depression	F20.4; F31.3-F31.5; F32.x; F33.x; F34.1; F41.2; F43.2
Amyloidosis	E85.x
Hyponatremia	E87.1
Hyperkalemia	E87.5
Mechanical ventilation	5A09357; 5A09457; 5A09557; 5A1945Z; 5A1955Z; 5A1935Z
Vasopressor medication	3E030XZ; 3E033XZ; 3E040XZ; 3E043XZ; 3E050XZ; 3E053XZ; 3E060XZ; 3E063XZ
Echocardiogram	B24xxxx
Coronary artery bypass surgery	021xxxx
CADID	027xxxx
Mitra-clip	02UG3JZ
TAVI	02RF3xx; 02RG3xx; 02RH3xx; 02RJ3xx
Electrical cardioversion	5A2204Z
Dialysis	5A1D xxx
Red cell transfusion	30230N0

CADID coronary artery dilatation with an intraluminal device. TAVI Trans-catheter aortic valve implantation

Supplementary Table S2. Absolute standardized differences before and after Propensity Score Matching (PSM) for men and women with T2DM matched with men and women without T2DM and for T2DM men matched with T2DM women hospitalized with heart failure with reduced ejection fraction in Spain (2016-2019)

	MEN WITH AND WITHOUT T2DM		WOMEN WITH AND WITHOUT T2DM		MEN AND WOMEN WITH T2DM	
	ASD before PSM	ASD after PSM	ASD before PSM	ASD after PSM	ASD before PSM	ASD after PSM
Age	-0.78	-0.07	-1.04	0.29	-7.89	-0.33
Number of conditions included in the CCI	22.61	0.71	29.87	0.01	41	2.78
High blood pressure, n (%)	Not included	Not included	Not included	Not included	-18.13	-3.55
COPD	16.35	2.82	25.16	9.78	Not included	Not included
Atrial fibrillation, n (%)	Not included	Not included	-1.55	1.07	Not included	Not included
Depression	4.18	2.18	2.10	0.81	Not included	Not included
Amyloidosis,	Not included	Not included	8.33	0.01	Not included	Not included

CCI; Charlson Comorbidity Index. Only variables used to estimate the propensity score are shown in the table. ASD Absolute Standardized Difference is shown as a percentage
